# How do men differ from women? Case-Control study on clinic and personality characteristics of eating disorders

**DOI:** 10.1192/j.eurpsy.2023.1800

**Published:** 2023-07-19

**Authors:** F. Ruiz Guerrero, J. Gonzalez Gómez, C. Cobo Gutierrez, L. Castro Fuentes, C. Hernández Jimenez, J. Romay González, A. Gómez del Barrio

**Affiliations:** 1 Psychiatry, Eating Disorders Unit, Hospital Universitario Marqués de Valdecilla, Santander, Spain

## Abstract

**Introduction:**

A review of the literature shows how female sex is a crucial factor in the development of ED, being the proportion of women and men 10 to 1 regardless of the location of the sample (Duncan, Ziobrowski & Nicol, 2017) and different clinical subtypes (AN, BN) (Swanson et al., 2011). However, male population has always been less studied, some works find that only 1% of the articles published in AN is aimed at the study of males (Galusca, 2012).

Nowadays it is accepted that the etiopathogenesis of these disorders is multifactorial and in addition to female gender other risk factors have been identified, such as neurobiological alterations, psychological predictors, personality traits, low self-esteem, extreme perfectionism or thinness values focused on body and figure. On the other hand, certain impulsive behaviours such as self-harm, substance use, physical activity or diets are factors that may be confused as predisposing or as symptoms of the pathology itself (Connan et al., 2003, Treasure, Stein and Maguire, 2015).

Recently, Kinasz, Accurso, Kass and Le Grange (2016) have compared the clinical characteristics that differentiate men (59) from women (560) in a sample of children and adolescents between 6 and 18 years-old, finding that males presented an earlier start of the ED and not appreciating differences in the duration of the disease, income, episodes of purgue and psychiatric comorbidity of anxiety, behaviour disorders or impulsivity.

**Objectives:**

The aim of this study was to evaluate gender differences in clinical characteristics, levels of depression, previous obsessiveness and personality dimensions in eating disorders (ED) compared with controls.

**Methods:**

A total of 80 participants was divided into 4 groups, 20 men and 20 women with ED and 20 men and 20 women without ED (healthy control), matched by age and socioeconomic status. The design of the study was case-control, and data was collected through clinical interview and a battery of cuestionaires.

**Results:**

Men with ED only differ in vigorous physical activity (measured by IPAQ) from controls and women with pathology. Regarding personality traits, men and women with ED do not differ among them, although they do differ in novelty search and harm avoidance respect to their controls.

**Conclusions:**

Behaviors such as physical activity in males frame a slightly different way of reducing their discomfort, however, clinical implication indicates that the treatment may be similar according to gender.

**Disclosure of Interest:**

None Declared

